# Charge Tethering Drives Intermediate-Range Order and Slow Dynamics in Zwitterionic Liquids

**DOI:** 10.1021/acs.jpcb.6c03524

**Published:** 2026-07-10

**Authors:** Raphael Ogbodo, Laxmi Adhikari, Clinton Adu, Andrew J. Nieuwkoop, Gary A. Baker, James F. Wishart, Claudio J. Margulis

**Affiliations:** † Department of Chemistry, 4083 The University of Iowa, Iowa City, Iowa 52242, United States; ‡ Department of Chemistry, 116043 University of Missouri, Columbia, Missouri 55211, United States; § Department of Chemistry and Chemical Biology, 5970 Rutgers University, Piscataway, New Jersey 08854, United States; ∥ Chemistry Division, 242612 Brookhaven National Laboratory, Upton, New York 11973-5000, United States

## Abstract

We have synthesized and characterized a family of zwitterionic liquids (ZwLs) based on the poly­(ethylene oxide) imidazolium cation and the alkyl sulfonate anion. To better rationalize ZwLs′ structural behavior and network-forming properties, we have computationally studied them in contrast to isostructural but chemically untethered ionic liquid (IL) analogues. Perhaps surprisingly, it is the molecular liquids and not the ILs that show the most self-assembly and intermediate-range order associated with scattering prepeaks; the ZwLs display also the slowest dynamics. This is consistent with prior work showing the importance of networks in considering the viscoelastic relaxation of liquids. We find that, because of their significantly large molecular dipoles, these zwitterions are versatile network-building blocks that result in highly viscous liquids. From a structural perspective, networks in these ZwLs sit somewhere in between what would be true chemical connectivity and the Coulombic networks observed in ionic liquids.

## Graphical abstract

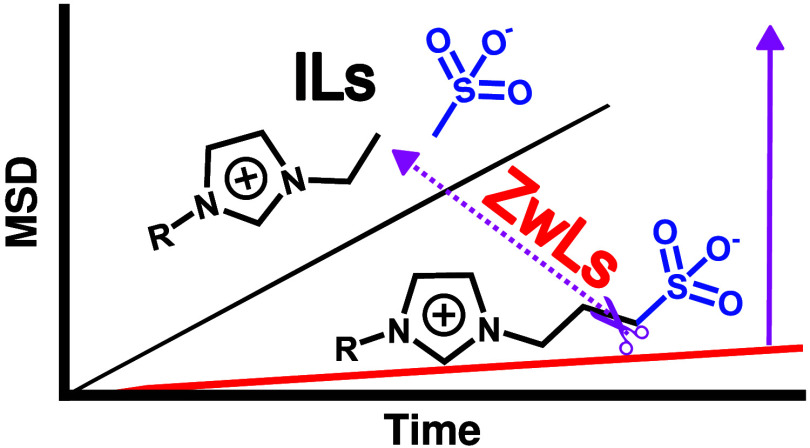

## Introduction

Zwitterions are intrinsically neutral molecules with large dipole moments. These form fascinating liquid systems with very high dielectric constants. Such media can be advantageous in electrochemical systems^1,2^ and find uses in biomedical applications as modifiers of nanoparticles for drug delivery^3^ and can even dissolve complex, recalcitrant biopolymers.^4^ They are the tethered dipolar relatives of the ionic liquids and, depending on their design, control can be had on the intramolecular separation between positive and negative charges and on their nondipolar tails. Whereas prior studies of similar zwitterionic liquids have focused primarily on applications and dielectric relaxation properties, the present work examines the structural features that distinguish them from their closely related counterparts, ionic liquids.

Figure 1 shows the molecular structures of ZwLs studied in this article and a set of computational IL analogues that we intend to contrast them with; conventions for naming, and molecular or ionic regions are highlighted as we refer to those in subsequent analysis. Because the computational analogues were devised with the goal of isolating and highlighting the effect of charge tethering on these systems without introducing further variables, we selected force field parameters for the ILs that are a very close match to those of the ZwLs within the obvious constraint of maintaining charge neutrality in the condensed phase; parameters are provided as Supporting Information. In other words, our goal was not to get the best true force field for the ILs but instead to get a force field that made them as close as possible to the ZwLs.

**Figure 1:**
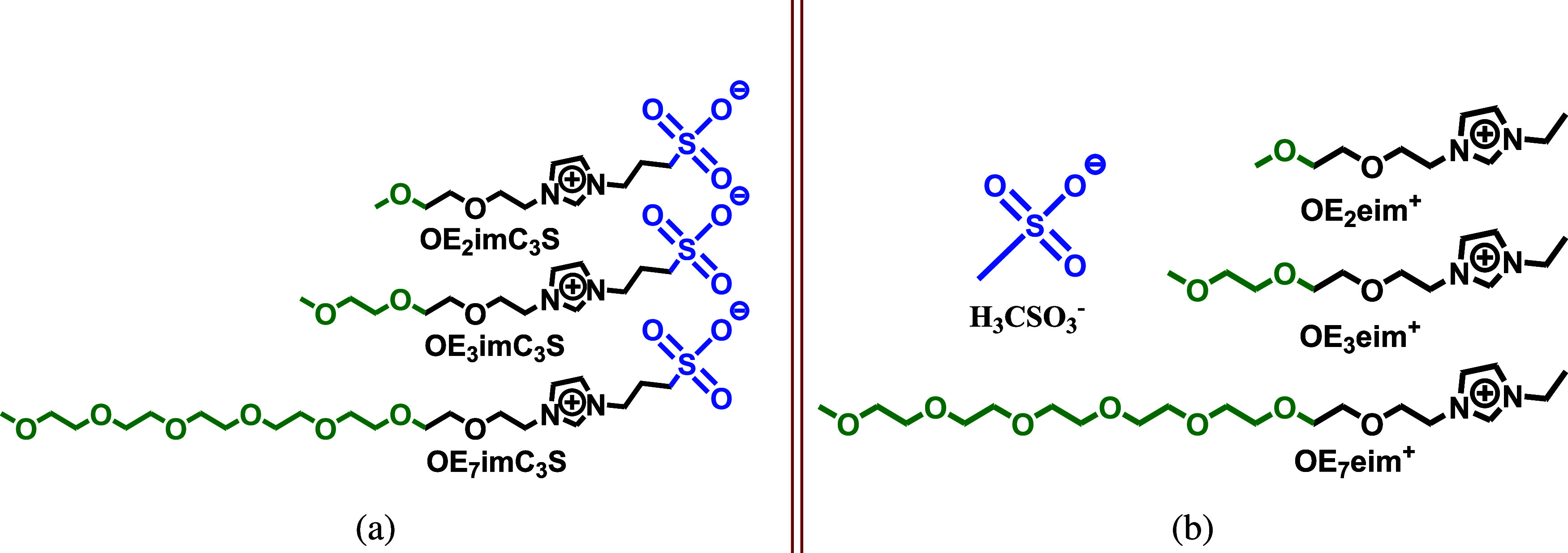
(a) Structure of ZwLs; color in this figure defines subcomponents used in the calculation of the partial subcomponents of *S*(*q*) in Figure 5; cationic component (black), anionic component (blue), and tail component (green). Naming conventions are shown below each structure; (b) isostructural ILs studied computationally.

## Methods

This section provides details on the characterization of the ZwLs and also provides computational methodological aspects of the study. NMR spectra (Figures S1–S3), densities (Table S1), differential scanning calorimetry thermograms (Figures S4–S6), and glass transition temperatures (Table S2), as well as details on the synthesis of ZwLs, are all provided in the SI.

### Physicochemical Properties Measurement for the Synthesized ZwLs

#### Density

Density was measured using 2 mL Corning Gay-Lussac density bottles (item 1622) from Corning Incorporated (Corning, NY). Temperatures of 293–313 K were achieved in 5 K increments using an 18 L Fisherbrand Mini Low Temperature Refrigerated Incubator (Catalog No.15–015–2632). Samples were equilibrated at each temperature for at least 15 min. Prior to each measurement, any liquid droplet exuded from the capillary bore due to thermal expansion was carefully removed using a Kimwipe. The bottle was calibrated using ultrapure water, and the final data were corrected with a temperature-dependent calibration factor. The measured and simulated densities for the three ZwLs are presented in Table S1.

#### Differential Scanning Calorimetry

Differential scanning calorimetry (DSC) was performed using a TA Instruments Q200 DSC with the Refrigerated Cooling System (RCS). 10–12 mg of previously dried sample was DSC weighed into an aluminum dsc pan, cooled to −98 °C, and isothermed for 4 min. The samples were gradually heated at 5 °C/min to 60 °C for OE_7_imC_3_S, and to 100 °C for OE_2_imC_3_S and OE_3_imC_3_S, under a nitrogen atmosphere. The data were analyzed using TA Instruments Trios v5 software. The DSC curves are shown in Figures S4–S6. The onset glass transition temperature is shown in Table S2. The glass transition onset temperatures (T_*g*_) were between a narrow range of −28.6 °C for OE_3_imC_3_S and −32.0 °C for OE_2_imC_3_S with OE_7_imC_3_S in between. There seems to be no any apparent trend in T_*g*_ (onset) across the different liquids.

#### Viscosity

Viscosities were determined using a Brookfield DV-III Ultra Programmable Rheometer (cone–plate viscometer) using 1 mL sample volume. The temperature of samples was controlled to within ± 0.05 K using a thermostatic water bath (Fisher Scientific Isotemp). Cannon at 298 ± 0.05 K was used as a certified viscosity standard.

### Computational Methods

Molecular dynamics simulations were all carried out using the GROMACS software version 5.1.4.^5^ The ZwLs and the IL analogues were modeled using force field parameters for Lennard-Jones, bonds, angles, and torsions which are based on the OPLS-AA,^6^ Canongia Lopes & Pádua,^7–10^ Price et al.,^11^ and Shimizu et al.^12^ The bonds, angles, and torsions parameters involving the oxygen atoms were taken from OPLS-AA,^6^ Weiner,^13^ and Cornell et al.^14^ The C–C–O–C and O–C–C–O torsion parameters were taken from Anderson and Wilson.^15^ Whereas most charges were derived from the aforementioned force fields, the charges on the methyl group in H_3_CSO_3_^–^ and those on the methylene group attached to SO_3_^–^ in the ZwLs were chosen so that the net charge of the negative groups displayed in blue in Figure 1 add up to –1. All parameters are provided as separate files in the Supporting Information.

The cutoff for all of the nonbonded interactions (Lennard-Jones and electrostatic) was set to 1.5 nm. The particle mesh Ewald^16,17^ method was used for the long-range electrostatic interactions with a sixth-order interpolation and Fourier spacing of 0.08 nm. 3D periodic boundary conditions were used as coded in the GROMACS software. The equations of motion were integrated using a time step of 1 fs with the MD integrator.^18,19^ An initial cubic configuration box for each of the ZwLs and the IL analogues was packed using the PACKMOL^20^ software package and energy-minimized. In the case of the ZwLs, each cubic configuration box contained 1000 molecules, whereas in the case of the ILs, each cubic configuration box contained 1000 ion pairs. Three successive equilibration steps were performed in the constant pressure, temperature, and number of particles (NPT) ensemble using the V-rescale thermostat^21^ and the Berendsen barostat^22^ with 0.2 and 1.0 ps time constants, respectively. The first step was run for 0.2 ns at a pressure of 50 bar and scaled partial charges of 1% of their nominal values; the second step was 2 ns in duration and was run at a pressure of 50 bar and scaled partial charges of 10%, and the third step was run for 2 ns at a pressure of 1 bar and 100% partial charges. After these equilibration steps, we ran 14 ns of simulated annealing in the NPT ensemble where the temperature was ramped up from 300 to 700 K and later cooled to 298 K. During the cooling protocol, snapshots were obtained as a function of temperature and were used as initial conditions for production runs. Whereas simulations discussed in the main text were at 600 K, we also generated production runs for the ZwLs at 298, 450, 500, and 700 K to produce the data shown in Table S1 and Figure S7. In each case, final production runs in the NPT ensemble were 10 ns in duration, except that for the specific purpose of calculating mean square displacements (MSDs) at 600 K for ILs and ZwLs (Figure 2), longer 60 ns production simulations were run in the NPT ensemble. Both the annealing and the production runs were carried out using the Nosé-Hoover thermostat^23^ and Parrinello–Rahman barostat^24^ with 0.2 and 1.0 ps time constants, respectively.

**Figure 2:**
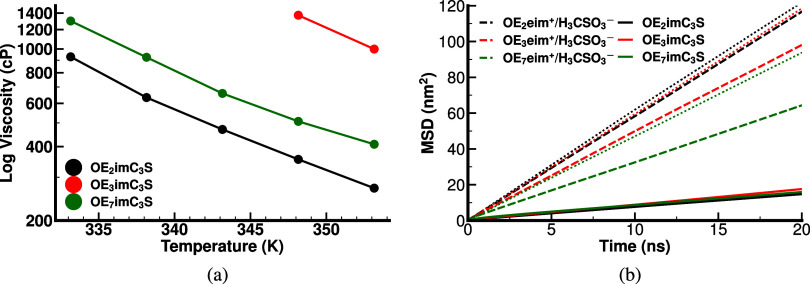
(a) Experimental viscosity and (b) computationally computed mean square displacement at 600 K for ZwLs and ions in matching ILs. ZwLs data represented with solid lines, cationic data shown with dashes and anionic data with dots. Data shown up to 20 ns was averaged over 60 ns.

The structure functions *S*(*q*) were computed from the last 2 ns of the 10 ns production runs using the equation

1 $$S\left(q\right)=\frac{\rho_{0}\sum_{i}\sum_{j}x_{i}x_{j}f_{i}\left(q\right)f_{j}\left(q\right)}{[\sum_{i}x_{i}f_{i}\left(q\right){]}^{2}}\int_{0}^{L/2}4\pi r^{2}\left(g_{\mathrm{ij}}\left(r\right)-1\right)\left(\frac{\sin\mathrm{qr}}{\mathrm{qr}}\right)W\left(r\right)dr$$

In eq 1, *x*_*i*_ and *x*_*j*_ are the atomic mole fractions of species *i* and *j* respectively, *g*_*ij*_(*r*) is the radial distribution function, ρ_*o*_ is the total number density, *f*_*i*_(*q*) and *f*_*j*_(*q*) are corresponding X-ray atomic form factors, and *W*(*r*) is a Lorch-type function^25,26^ used to minimize the effect of pair distribution function truncation due to finite box sizes. In the Results and Discussion section, partial subcomponents of *S*(*q*) are displayed; such partitions have already been described in multiple prior publications.^27–46^

The magnitude of the dipole moment for zwitterion *i*_mol_ has a value

2 $$\mu_{i_{\mathrm{mol}}}=\left|\sum_{j_{\mathrm{at}}}q_{i_{\mathrm{mol}},j_{\mathrm{at}}}\cdot r_{i_{\mathrm{mo}l},j_{\mathrm{at}}}\right|$$

and the average magnitude of a molecular dipole over a simulation snapshot is

3 $$\mu =\frac{\sum_{i_{\mathrm{mol}}}\mu_{i_{\mathrm{mol}}}}{N}$$

where *N* is the total number of molecules. In Figures 6 and S7, the distribution of dipole magnitudes and corresponding averages are computed over a 10 ns period.

## Results and Discussion

The differential scanning calorimetry thermograms for the three zwitterionic liquids in Figures S4–S6 do not show any features attributable to melting or freezing under the dynamic conditions of the scans, which is unsurprising considering that their very high viscosities result in slow crystallization kinetics that make them easily trapped in the glassy state when cooled. Their glass transition onset temperatures in Table S2 fall within the narrow range of −32.0 to −28.6 °C. The very high measured viscosities of these zwitterionic liquids (see Figure 2a) are connected to their relatively high glass transition onset temperatures. Consistent with this, OE_3_imC_3_S has the highest T_*g*_ and the highest viscosity of the three liquids over the observed temperature range. While there is an apparent nonmonotonic effect of ether chain length on the dynamical properties of these liquids, that effect is small compared to the effect of tethering the cationic and anionic groups together. For a rough comparison with ionic liquids containing the same ionic centers, the T_*g*_ of 1-ethyl-3-methylimidazolium methanesulfonate is −68 °C^47^ and the estimated T_*g*_ of 1-butyl-3-methylimidazolium methanesulfonate is −59 °C.^48^

Because ZwLs are highly viscous (their viscosity at room temperature is on the order of 10^3^–10^4^ cP as can be derived from Figure 2a), meaningful simulations had to be run at a significantly high temperature. Figure 2b highlights that, even at 600 K, ZwLs do not exit their cage regime for many nanoseconds; this can be gleaned from a comparison between the square root of the MSD and the typical nanometer length scale of the ZwLs. On the same time scale, and at the same temperature, cations and anions in the IL analogs translate multiple times their size.

When poly­(ethylene oxide) tails are long, as in the case of OE_7_imC_3_S and OE_7_eim^+^/H_3_CSO_3_^–^, structural differences between the ZwL and the corresponding IL are striking and easily discernible from simulation snapshots as can be gleaned from Figure 3a,b. OE_7_imC_3_S displays thick and well-formed tail domains that are segregated from dipole agglomerates; in contrast, wherereas charge networks and tail domains are present in the case of the OE_7_eim^+^/H_3_CSO_3_^–^ ionic liquid, they are significantly less well-defined. Figure S8a,b shows that differences become more subtle when tails are shorter.

**Figure 3:**
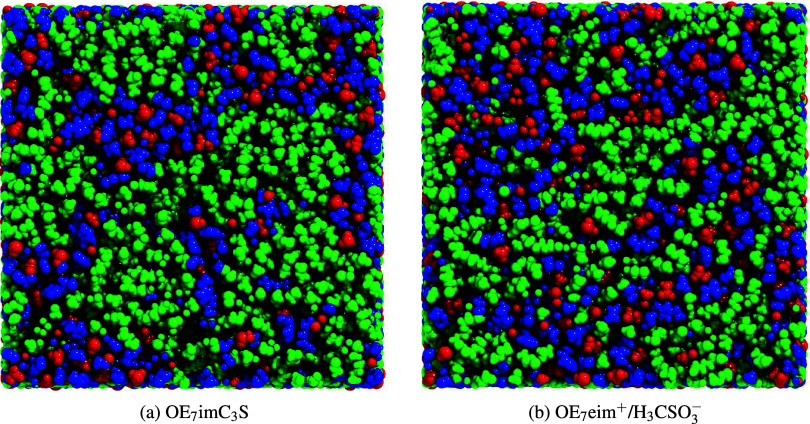
Simulation snapshot for OE_7_imC_3_S (a) and its IL analogue OE_7_eim^+^/H_3_CSO_3_^–^ (b). Figures show characteristic nanoscale domain segregation within the liquid structure being significantly more notable in the case of the dipolar ZwL than the ionic liquid. In the Figure, tail components are depicted in green, cationic head components in blue, and anionic components in red.

What Figure 3 shows qualitatively can be quantitatively discussed in the context of scattering. Figure 4 shows the computed X-ray *S*(*q*) for each liquid. Matching what is observed in Figure 3, OE_7_imC_3_S shows a massive first sharp diffraction peak below 0.5 Å^–1^, whereas OE_7_eim^+^/H_3_CSO_3_^–^ only shows a modest prepeak in the same *q*-region. This peak defines intermediate range order and, consistent with Figure 3, there is significantly more tail-domain – charge-aggregate separation in the case of OE_7_imC_3_S. Notice that the prepeak size pattern persists for OE_3_imC_3_S when compared with OE_3_eim^+^/H_3_CSO_3_^–^, but there is much less of a difference in *S*(*q*) when comparing OE_2_imC_3_S with its IL analog OE_2_eim^+^/H_3_CSO_3_^–^.

**Figure 4:**
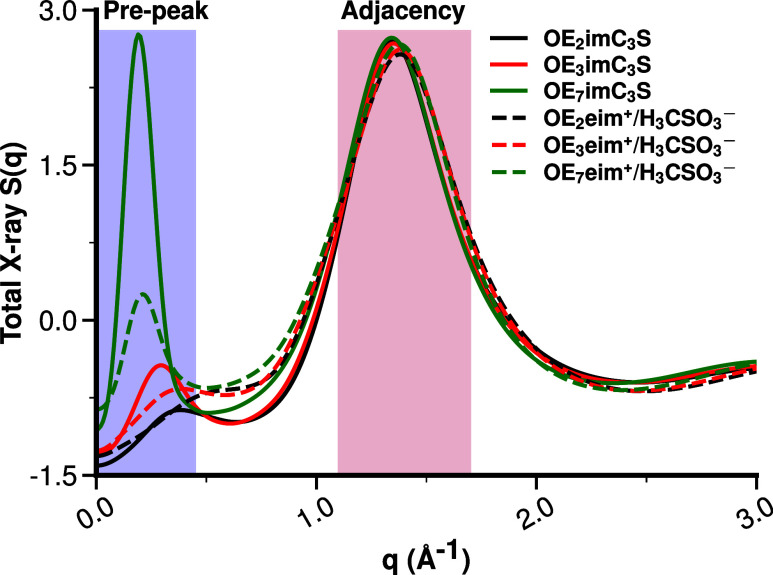
Simulated total X-ray structure function, *S*(*q*), for the ZwLs (solid lines) and the IL analogues (dashed lines) at 600 K. The prepeak, or first sharp diffraction peak (FSDP), is attributed to pronounced nanoscale structural segregation between well-defined charge-rich, or dipole-rich, domains and the poly­(ethylene oxide) tails. Consistent with Figure 3, the level of intermediate-range order associated with the prepeak is massively more prominent in the case of ZwLs with sufficiently long tails when compared to the analogous ILs. Notice also the lack of charge alternation peak at around 0.8 Å^–1^ and the discussion regarding this point associated with Figure 5.

A notable finding is that, in all of these systems, the hallmark feature capturing charge alternation is missing in *S*(*q*).^33–46^ This is not uncommon; because of lack of contrast in X-ray experiments, many ILs do not show a charge alternation peak which in this case should appear at around 0.8 Å^–1^. The charge alternation pattern is certainly present in the liquid phase as can be glean from Figure 3a,b (and from Figure S8a,b); moreover, it is clearly discernible in peaks and antipeaks at around 0.8 Å^–1^ for partial subcomponents of *S*(*q*) in Figure 5. In the case of OE_7_imC_3_S, the partial subcomponents of *S*(*q*) show very large first sharp diffraction peaks and antipeaks below 0.5 Å^–1^. These reveal how pronounced the dipole agglomerate – poly­(ethylene oxide) alternation pattern is in the liquid phase, or put differently, how significant intermediate range order is for this system. Specifically, examine the very large head–tail and tail–anion antipeaks below 0.5 Å^–1^ and positive-going peaks in the same *q*-region for anion–anion, head–anion, head–head, and tail–tail subcomponents. We have explained in prior publications^27–46^ that positive-going peaks in this region correspond to same type (in this case, either charged–charged independent of sign, or poly­(ethylene oxide)-poly­(ethylene oxide)) correlations, whereas negative-going peaks correspond to opposite-type (charged-poly­(ethylene oxide)) interactions. The same type of logic applies to correlations at 0.8 Å^–1^. In this case, same type means positive-positive and negative-negative charge correlations, whereas opposite type means positive–negative correlations. Same charge-type correlations result in peaks at 0.8 Å^–1^, whereas opposite-type correlations lead to antipeaks (see anion–anion, head–anion, and head–head subplots within Figure 5).

**Figure 5:**
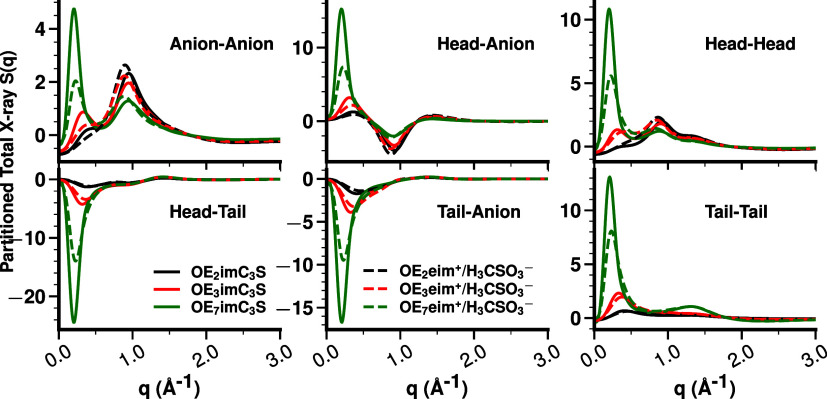
Partial subcomponents of the total X-ray structure function *S*(*q*). Atoms in the head, anion, and tail are as defined in Figure 1. Structural correlations for the ZwLs (solid lines) and IL analogues (dashed lines) all are computed at 600 K.

The reader may reasonably ask what makes these ZwLs such good intermediate-range order builders. We believe that it may be, at least in part, intramolecular structural frustration and their inability to coil charge against charge. Figure 6 shows a few remarkable things: (1) the distribution of dipoles is quite similar across all our studied ZwLs, (2) the coiled conformation in which the negative part of the dipole approaches the positive part has minimal probability, likely due to sterics, and (3) the most likely dipole values are large (compared with some biological zwitterions at around 15 D). Such large dipoles in zwitterionic liquids have also been reported recently.^1,2^ Furthermore, based on simulation results, these dipoles vary little with temperature in the range from 298–700 K (see Figure S7 in the SI).

**Figure 6:**
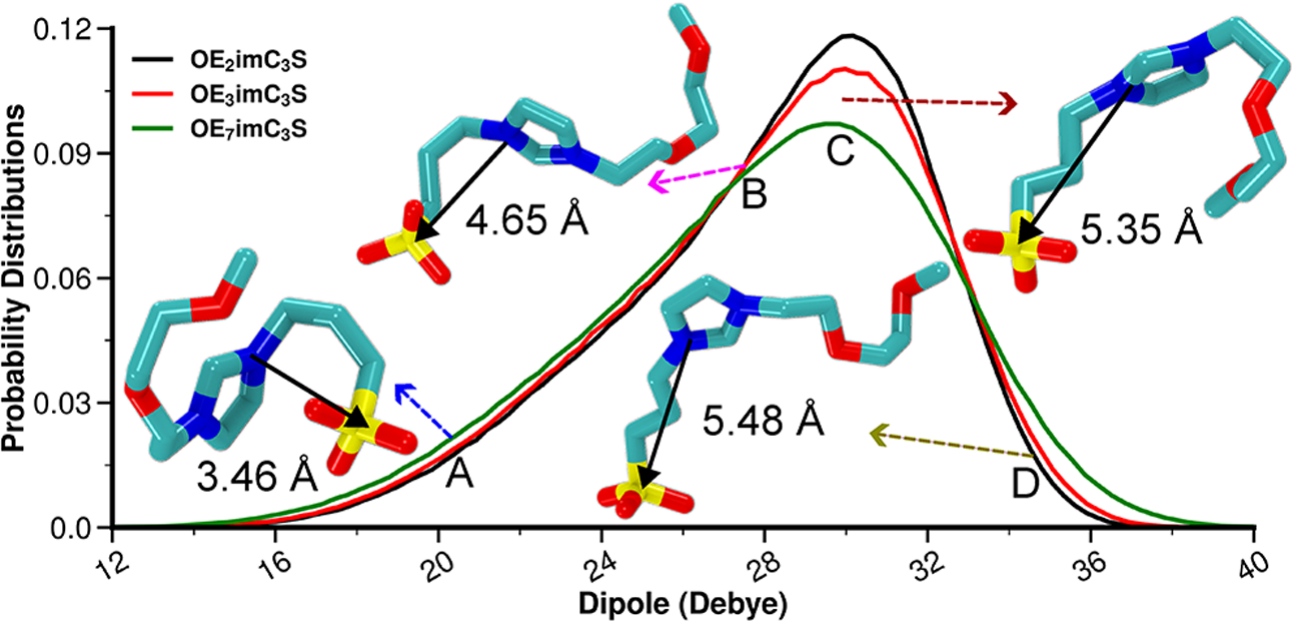
Computed dipole moment distributions for the ZwLs based on simulations at 600 K. Representative configurations are shown for the case of OE_2_imC_3_S, highlighting intramolecular distances. Dipole distributions are quite similar across the family of ZwLs.

This apparent structural frustration, a feature of the molecular bridging portion separating positive and negative parts within these ZwLs, can result in all of the buildup and stacking patterns for the dipoles depicted in Figure 7. The pattern labeled B in the figure (the antiparallel dipole configuration) is very common for ZwLs but not for their IL analogues. Pattern A is very common for both the ZwLs and ILs.

**Figure 7:**
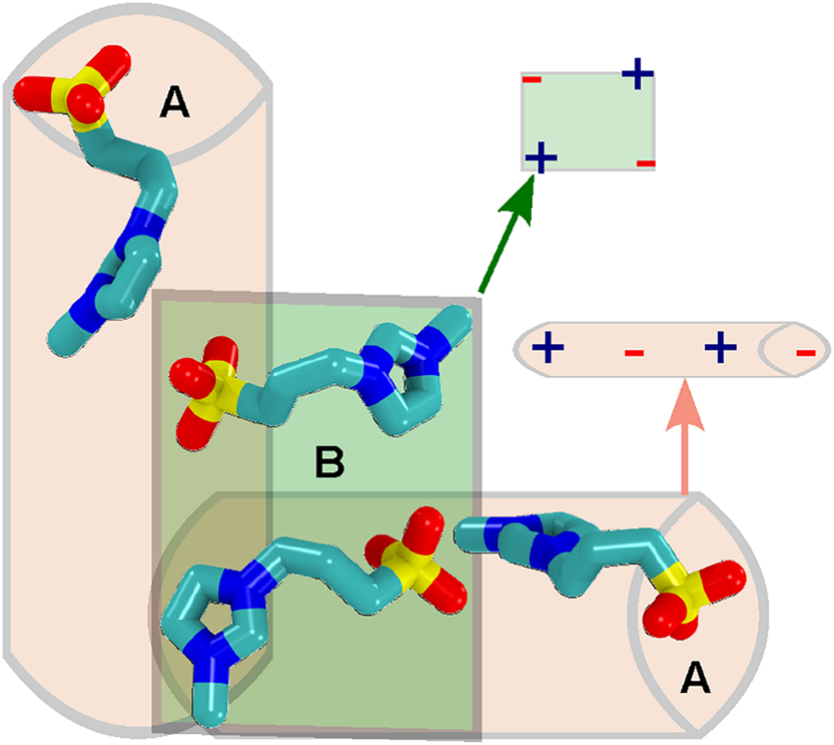
This figure illustrates possible interaction patterns observed for ZwLs. Whereas A and B style patterns are common for ZwLs, only A is common in the charge networks of ILs. For clarity, cation tails have been omitted from this depiction. In the case of ZwLs with longer poly­(ethylene oxide) chains, the charge network stacking appears to promote also greater segregation of the tail domains, leading to the formation of thicker nanoscale regions compared to the IL analogues. This is evident in the simulation snapshots shown in Figure 3 and *S*(*q*) functions shown in Figures 4 and 5.

In a recent article, we compared prototypical imidazolium-based ILs with otherwise analogous but OH-tail-terminated cations all coupled with the same anions.^49^ In that case, we found that the OH-terminated types were significantly more sluggish than the nonhydroxylated counterparts, and interestingly, the hydroxylated ILs lacked the prepeak and intermediate range order that prototypical ILs with alkyl tails display at low q. In contrast, when comparing ZwLs with analogous ILs, we find that it is the ZwLs that have the more significant intermediate range order and sluggishness. In other words, the presence or absence of intermediate-range order does not appear to be the primary factor governing slow dynamics. Instead, charge networks, or in the case of ZwLs, charge alternation and charge stacking, appear to be tremendously important. This is consistent with prior articles^50–53^ showing that the majority of the viscoelastic relaxation in ILs is due to charge network dynamics and not to the dynamics of the prepeak associated with intermediate range order.

## Conclusions

This article is one in a sequence that studies the charge connectivity and mobility of certain IL-related materials. It is clear from these studies that a hierarchy emerges for materials progressing from the polymerized ILs to ZwLs, then to OH-terminated ILs, and finally to the most prototypical “canonical” ILs. Even though ZwLs are neutral molecules, it is better to think of them as tethered ions that, because of bonding constraints, must stay separated and form significantly large dipoles that do not change significantly with temperature. What appears to dominate the sluggishness of the materials in the aforementioned hierarchy is the dynamics of the charge network and the manner in which moieties can tether chemically or physically to or within it. In particular, ZwLs can typically adopt an antiparallel conformation that adds to the arsenal of arrangements charge assemblies can take; hydroxyl tail-terminated ILs can also become more sluggish when OH groups can participate in interactions with the charge network. Seen as a whole, the hierarchy associated with liquid sluggishness appears to favor those motifs in which there is strong chemical or physical participation with the charge network.

## Supplementary Material





## Notes

Details on the synthesis of ZwLs; ^1^H NMR spectra; thermograms; tables with densities and glass transition onsets; graph with average temperature-dependent dipole moments; and a simulation snapshot for OE_2_imC_3_S and its IL analogue OE_2_eim^+^/H_3_CSO_3_^–^ (PDF)
Gromacs topology files (ZIP)
