# Supplementary material for: Charge Tethering Drives Intermediate-Range Order and Slow Dynamics in Zwitterionic Liquids
Source: J Phys Chem B. 2026 Jul 10;130(29):7444–50. doi: 10.1021/acs.jpcb.6c03524 (PMC13403304; doi:10.1021/acs.jpcb.6c03524)
Supplement: Supplementary file 1 [file jp6c03524_si_001.pdf]

Charge Tethering Drives Intermediate-Range Order and Slow Dynamics in Zwitterionic Liquids

Raphael Ogbodo,[†] Laxmi Adhikari,[‡] Clinton Adu,[¶] Andrew J. Nieuwkoop,[*,¶] Gary A. Baker,[*,‡] James F. Wishart,[*,§] and Claudio J. Margulis[*,†]

†*Department of Chemistry, The University of Iowa, Iowa City, IA 52242, United States*

‡*Department of Chemistry, University of Missouri, Columbia, MO 55211, United States*

¶*Department of Chemistry and Chemical Biology, Rutgers University, Piscataway, New Jersey 08854, United States*

§*Chemistry Division, Brookhaven National Laboratory, Upton, New York 11973-5000, United States*

E-mail: an567@chem.rutgers.edu; bakergar@missouri.edu; wishart@bnl.gov; claudio-margulis@uiowa.edu

Contents

S.1 Synthesis **S3**

S.1.1 Synthesis of zwitterionic surfactant: 3-(1-(2-(2-methoxyethoxy)ethyl)-1H-3-imidazol-1-ium-3-yl)propane-1-sulfonate, OE_2imC_3S. S3

S.1.1.1 Synthesis of 2-(2-methoxyethoxy)ethyl 4-methylbenzenesulfonate, OE_2TS. S3

S.1.1.2 Synthesis of 1-(2-(2-methoxyethoxy)ethyl)-1H-imidazole, OE_2im. S3

S.1.1.3 Synthesis of 3-(1-(2-(2-methoxyethoxy)ethyl)-1H-3-imidazol-1-ium-3-yl)propane-1-sulfonate, OE_2imC_3S. S4

S.1.2 Synthesis of zwitterionic surfactant: 3-(1-(2-(2-(2-methoxyethoxy)ethoxy)ethyl)-1H-3-imidazol-1-ium-3-yl)propane-1-sulfonate, OE_3imC_3S. S4

S.1.2.1 Synthesis of 2-(2-(2-methoxyethoxy)ethoxy)ethyl 4-methylbenzenesulfonate, OE_3TS. S4

S.1.2.2 Synthesis of 1-2-(2-(2-methoxyethoxy)ethoxy)ethyl)-1H-imidazole, OE_3im. S4

S.1.2.3 Synthesis of 3-(1-(2-(2-(2-methoxyethoxy)ethoxy)ethyl)-1H-3-imidazol-1-ium-3-yl)propane-1-sulfonate, OE_3imC_3S. S5

S.1.3 Synthesis of zwitterionic surfactant: 3-(1-(2,5,8,11,14,17,20-heptaoxadocosan-22-yl)-1H-imidazol-3-yl)propane-1-sulfonate, OE_7imC_3S. S5

S.1.3.1 Synthesis of 2,5,8,11,14,17,20-heptaoxadocosan-22-yl 4-methylbenzenesulfonate, OE_7TS. S5

S.1.3.2 Synthesis of 1-(2,5,8,11,14,17,20-heptaoxadocosan-22-yl)-1H-imidazole , OE_7im. S6

S.1.3.3 Synthesis of 3-(1-(2,5,8,11,14,17,20-heptaoxadocosan-22-yl)-1H-imidazol-3-yl)propane-1-sulfonate, OE_7imC_3S. S6

S.2 Additional Tables and Figures **S7**

S.1 Synthesis

This section describes the synthesis of ZwLs studied in this article.

S.1.1 Synthesis of zwitterionic surfactant: 3-(1-(2-(2-methoxyethoxy)ethyl)-1H-3-imidazol-1-ium-3-yl)propane-1-sulfonate, OE_2imC_3S.

S.1.1.1 Synthesis of 2-(2-methoxyethoxy)ethyl 4-methylbenzenesulfonate, OE_2TS.

In a typical procedure, 10 g (0.083 mol) of diethylene glycol methyl ether was added to a solution of sodium hydroxide (9.56 g, 0.239 mol) dissolved in 50 mL of tetrahydrofuran (50% in water). The mixture was placed in an ice bath for 15 min, and 15.32 g (0.080 mol) of 4-toluene sulfonyl chloride dissolved in 50 mL of tetrahydrofuran was added dropwise over a period of 2 h under magnetic stirring. The reaction mixture was then poured into cold water and extracted three times with dichloromethane. The organic layers were collected and dried over magnesium sulfate and finally removed by rotary evaporation at 30 °C for 10 h to yield 19.75 g of OE_2TS as a colorless liquid (90% yield) which was used without further purification.

S.1.1.2 Synthesis of 1-(2-(2-methoxyethoxy)ethyl)-1H-imidazole, OE_2im.

A mixture of imidazole (5.11 g, 0.075 mol), NaOH (3.20 g, 0.08 mol) and 2-(2-methoxyethoxy)ethyl p-toluenesulfonate (19.50 g, 0.071 mol) in 40 mL of tetrahydrofuran was refluxed for 24 h. After cooling and removal of all solvents under vacuum, the reaction mixture was added to water and extracted three times with dichloromethane. The combined organic layer was dried over magnesium sulfate and finally removed by rotary evaporation at 50 °C for 6 h to obtain an imidazole derivative, OE_2im, as a colorless liquid (yield = 10.99 g, 91%).

S.1.1.3 Synthesis of 3-(1-(2-(2-methoxyethoxy)ethyl)-1H-3-imidazol-1-ium-3-yl)propane-1-sulfonate, OE_2imC_3S.

10.90 g (0.064 mol) of 1-(2-Methoxyethyl)-1H-imidazole was dissolved in 40 mL of acetonitrile under an argon atmosphere, and then 7.82 g (0.064 mol) of 1,3-propanesultone. The mixture was refluxed for 30 h, followed by rotary evaporation at 50 °C to remove the solvent. The residual liquid was washed several times with diethyl ether by decantation. Then, the product was dried in a rotary evaporator at 50 °C for 24 h to obtain OE_2imC_3S as a pale-yellow viscous liquid (yield 14.05 g, 75%). The ^{1}H NMR of the product is shown in Fig S1.

S.1.2 Synthesis of zwitterionic surfactant: 3-(1-(2-(2-(2-methoxyethoxy)ethoxy)ethyl)-1H-3-imidazol-1-ium-3-yl)propane-1-sulfonate, OE_3imC_3S.

S.1.2.1 Synthesis of 2-(2-(2-methoxyethoxy)ethoxy)ethyl 4-methylbenzenesulfonate, OE_3TS.

In a typical procedure, 10 g (0.061 mol) of triethylene glycol monomethyl ether was added to a solution of sodium hydroxide (6.82 g, 0.171 mol) dissolved in 50 mL of tetrahydrofuran (50% in water). The mixture was placed in an ice bath for 15 min, and 11.44 g (0.060 mol) of 4-toluene sulfonyl chloride dissolved in 50 mL of tetrahydrofuran was added dropwise over a period of 2 h under magnetic stirring. The reaction mixture was then poured into cold water and extracted three times with dichloromethane. The organic layers were collected and dried over magnesium sulfate and finally removed by rotary evaporation at 30 °C for 10 h to yield 15.7 g of OE_3TS as a colorless liquid (82% yield) which was used without further purification.

S.1.2.2 Synthesis of 1-2-(2-(2-methoxyethoxy)ethoxy)ethyl)-1H-imidazole, OE_3im.

A mixture of imidazole (3.48 g, 0.051 mol), NaOH (2.60 g, 0.065 mol), and 2-(2-(2-methoxyethoxy)ethoxy)ethyl 4-methylbenzenesulfonate (15.68 g, 0.049 mol) in 40 mL of tetrahydrofuran was refluxed for 24 h. After cooling and removal of all solvents under vacuum, the reaction mixture was diluted with water and extracted three times with dichloromethane. The combined organic layer was dried over

magnesium sulfate and finally removed by rotary evaporation at 50 °C for 6 h to obtain 8.71 g of imidazole derivative, OE_3im, as a colorless liquid (83% yield).

S.1.2.3 Synthesis of 3-(1-(2-(2-(2-methoxyethoxy)ethoxy)ethyl)-1H-3-imidazol-1-ium-3-yl)propane-1-sulfonate, OE_3imC_3S.

8.68 g (0.040 mol) of 2-(2-(2-methoxyethoxy)ethoxy)ethyl)-1H-imidazole was dissolved in 40 mL acetonitrile under an argon atmosphere and added with 4.95 g (0.040 mol) of 1,3-propanesultone. The mixture was refluxed for 40 h, followed by rotary evaporation at 50 °C to remove the solvent. The residual liquid was washed several times with diethyl ether by decantation. Then, the product was dried in a rotary evaporator at 50 °C for 24 h to obtain 10.20 g of OE_3imC_3S as a brown viscous liquid (76% yield). The ^{1}H NMR of the product is shown in Fig. S2.

S.1.3 Synthesis of zwitterionic surfactant: 3-(1-(2,5,8,11,14,17,20-heptaoxadocosan-22-yl)-1H-imidazol-3-yl)propane-1-sulfonate, OE_7imC_3S.

S.1.3.1 Synthesis of 2,5,8,11,14,17,20-heptaoxadocosan-22-yl 4-methylbenzenesulfonate, OE_7TS.

10.23 g (0.029 mol) of methoxypolyethylene glycol 350 was added to a solution of sodium hydroxide (3.37 g, 0.084 mol) dissolved in 50 mL of tetrahydrofuran (50% in water). The mixture was placed in an ice bath for 15 min, and 5.56 g (0.029 mol) of 4-toluene sulfonyl chloride dissolved in 50 mL of tetrahydrofuran was added dropwise over a period of 2 h under magnetic stirring. The reaction mixture was then poured into cold water and extracted three times with dichloromethane. The organic layers were collected and dried over magnesium sulfate and finally removed by rotary evaporation at 30 °C for 10 h to yield 11.24 g OE_7TS as a colorless liquid (76.3% yield) which was used without further purification.

S.1.3.2 Synthesis of 1-(2,5,8,11,14,17,20-heptaoxadocosan-22-yl)-1H-imidazole , OE_7im.

A mixture of imidazole (1.603 g, 0.023mol), NaOH (1.07 g, 0.027 mol), and OE_7TS (11.24 g, 0.022 mol) in 40 mL of tetrahydrofuran was refluxed for 24 h. After cooling and removal of all solvents under vacuum, the reaction mixture was diluted with water and extracted three times with dichloromethane. The combined organic layer was dried over magnesium sulfate and finally removed by rotary evaporation at 50 °C for 6 h to obtain an imidazole derivative, OE_7im, as a yellow liquid (yield = 7.65 g, 86.9%) which was used without further purification.

S.1.3.3 Synthesis of 3-(1-(2,5,8,11,14,17,20-heptaoxadocosan-22-yl)-1H-imidazol-3-yl)propane-1-sulfonate, OE_7imC_3S.

7.65 g (0.019 mol) of 1-(2-Methoxyethyl)-1H-imidazole was dissolved in 30 mL of acetonitrile under an argon atmosphere, and then 2.32 g (0.019 mol) of 1,3-propanesultone. The mixture was refluxed for 30 h, followed by rotary evaporation at 50 °C to remove the solvent. The reaction mixture was then added with dichloromethane and extracted three times with water. Then, the product was dried in a rotary evaporator at 50 °C for 24 h to obtain OE_7imC_3S as a brown viscous liquid (yield 8.0 g, 80.6%). The 1H NMR of the product is shown in Fig. S3.

S.2 Additional Tables and Figures

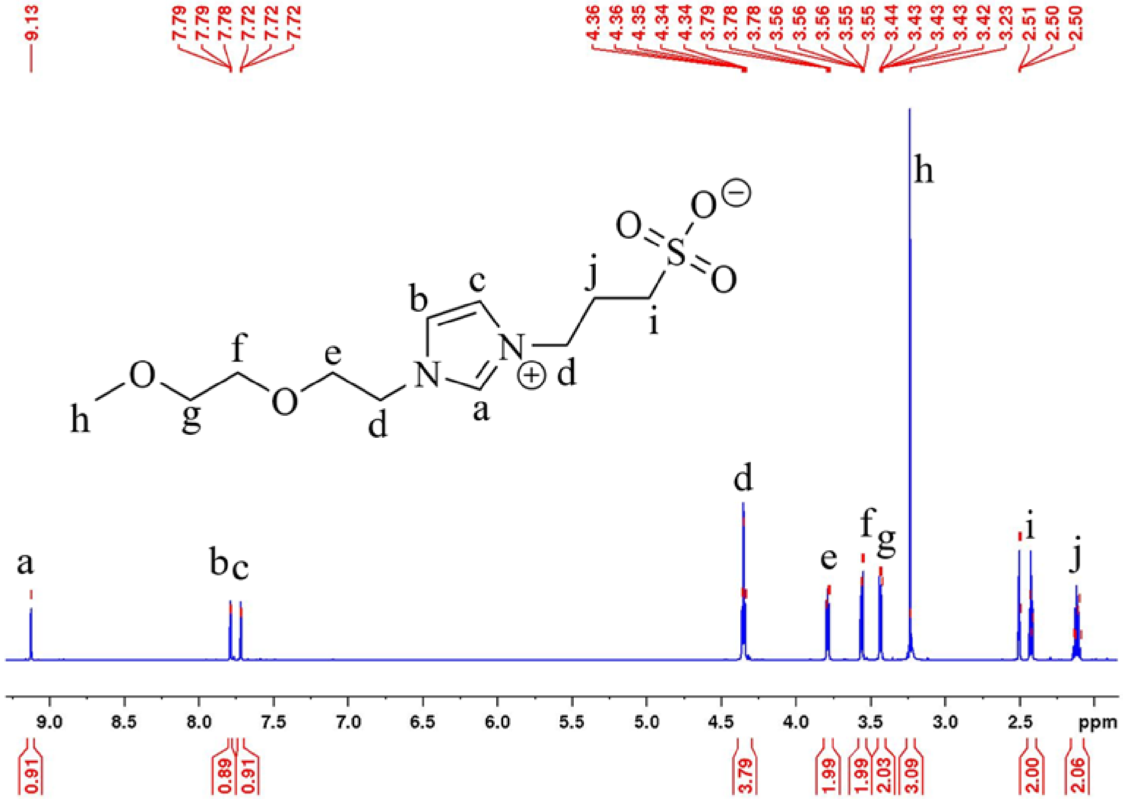

Figure S1: ^{1}H NMR spectrum of OE_2imC_3S: ^{1}H NMR (600 MHz, DMSO) δ 9.14 (s, 1H), 7.80 (t, J = 1.8 Hz, 1H), 7.74 (t, J = 1.8 Hz, 1H), 4.35–4.31 (m, 4H), 3.78–3.76 (m, 2H), 3.55–3.53 (m, 2H), 3.42–3.41 (m, 2H), 3.22 (s, 3H), 2.49 (dd, J = 7.6, 6.8 Hz, 2H), 2.14–2.04 (m, 2H).

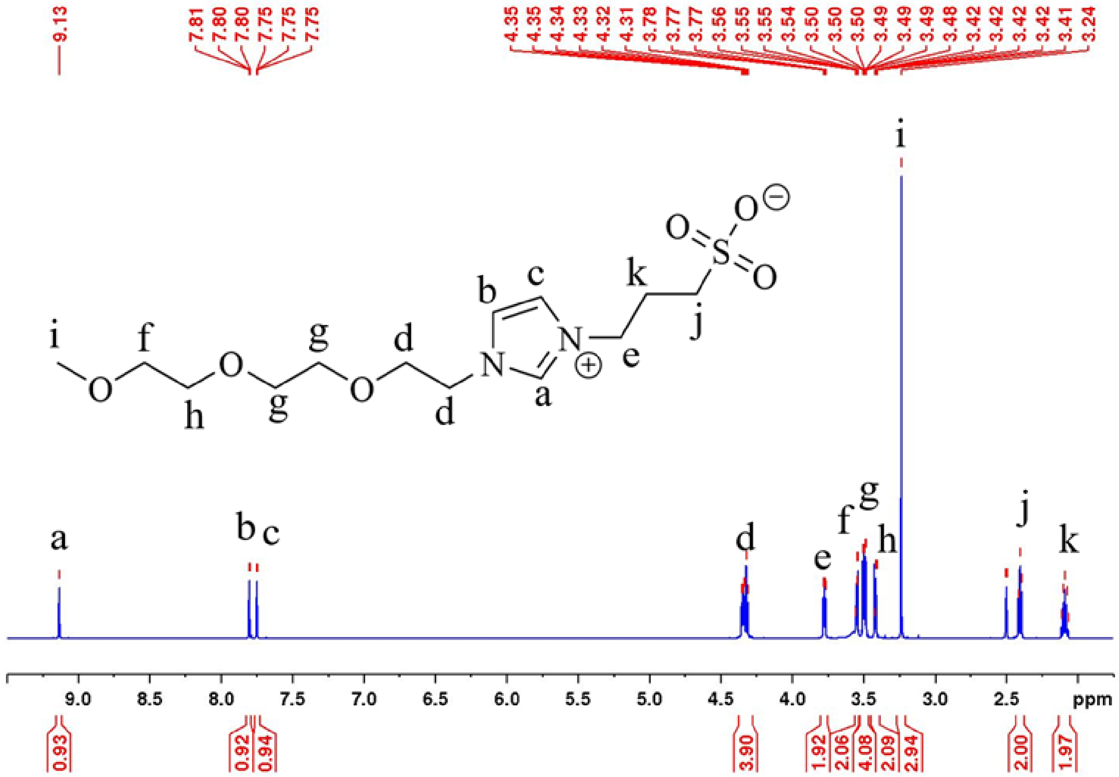

Figure S2: ^{1}H NMR spectrum of OE_3imC_3S: ^{1}H NMR (600 MHz, DMSO) δ 9.14 (s, 1H), 7.80 (t, J = 1.8 Hz, 1H), 7.74 (t, J = 1.8 Hz, 1H), 4.35–4.31 (m, 4H), 3.78–3.76 (m, 2H), 3.55–3.53 (m, 2H), 3.42–3.41 (m, 2H), 3.22 (s, 3H), 2.49 (dd, J = 7.6, 6.8 Hz, 2H), 2.14–2.04 (m, 2H).

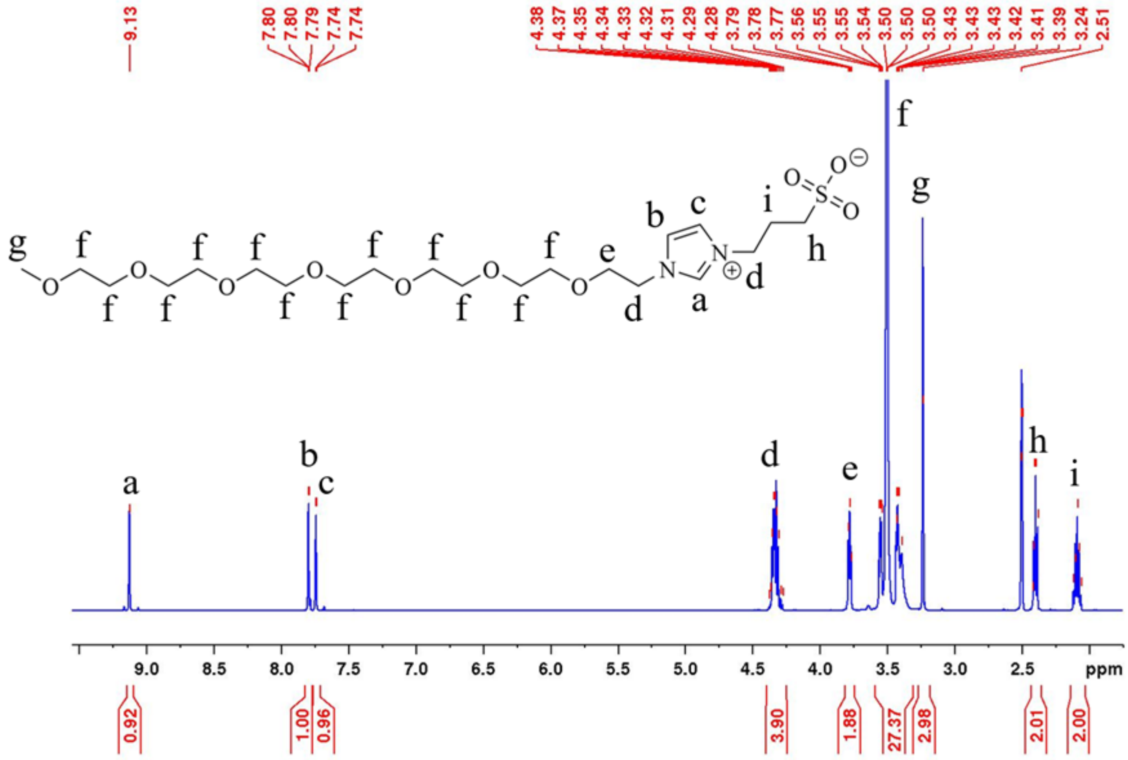

Figure S3: ^{1}H NMR spectrum of OE_7imC_3S: ^{1}H NMR (600 MHz, DMSO) δ 9.14 (s, 1H), 7.80 (t, J = 1.8 Hz, 1H), 7.74 (t, J = 1.8 Hz, 1H), 4.35–4.31 (m, 4H), 3.78–3.76 (m, 2H), 3.55–3.53 (m, 2H), 3.42–3.41 (m, 2H), 3.22 (s, 3H), 2.49 (dd, J = 7.6, 6.8 Hz, 2H), 2.14–2.04 (m, 2H).

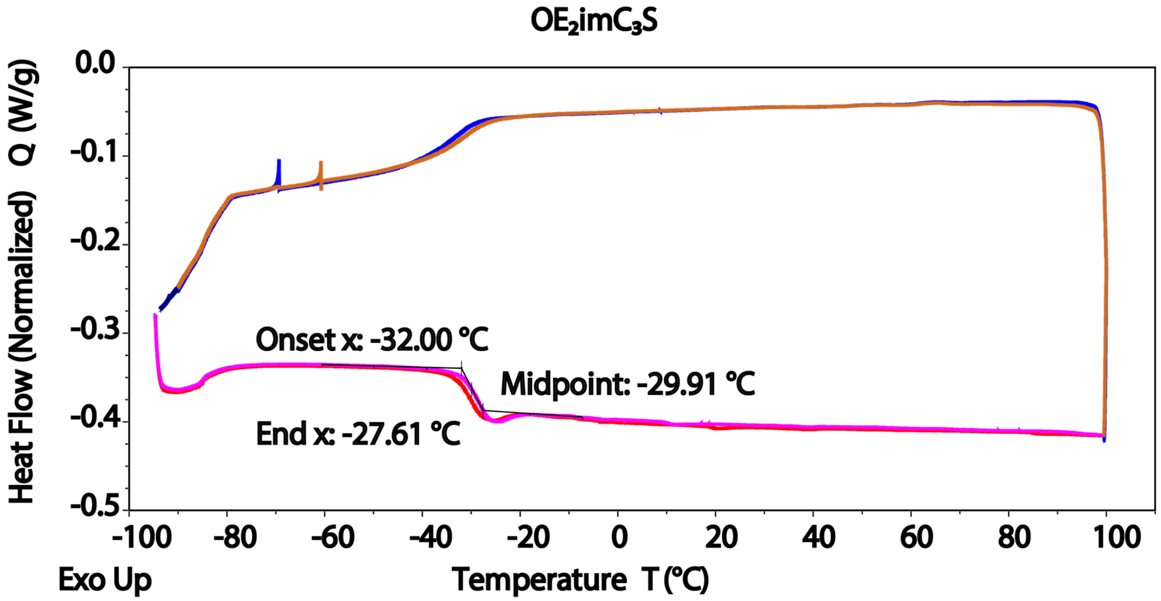

Figure S4: DSC thermogram for OE_2imC_3S.

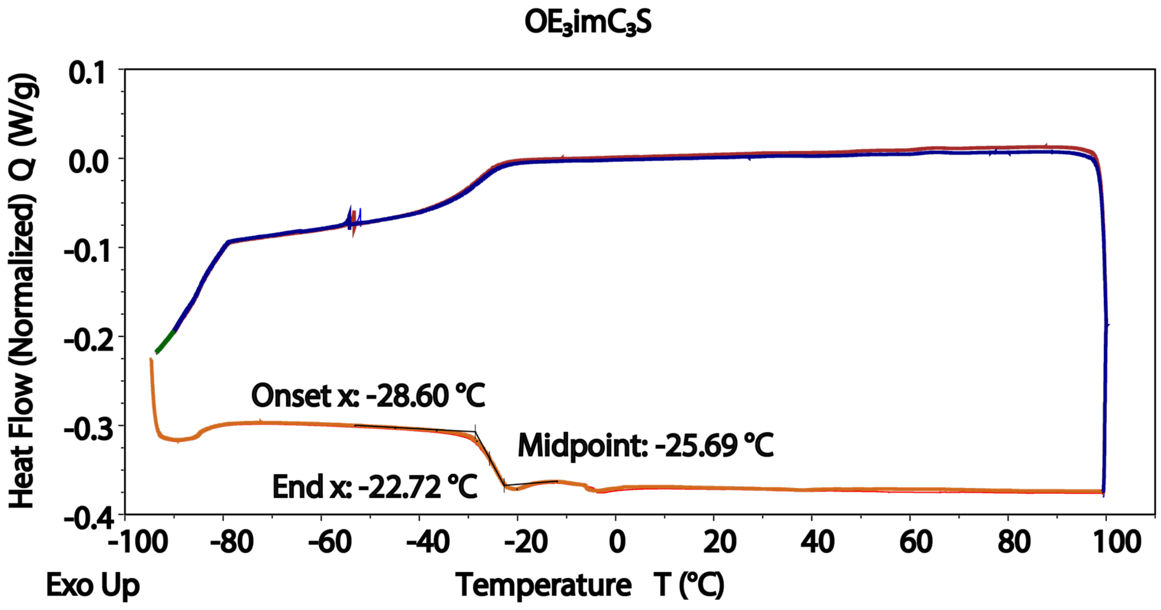

Figure S5: DSC thermogram for OE_3imC_3S.

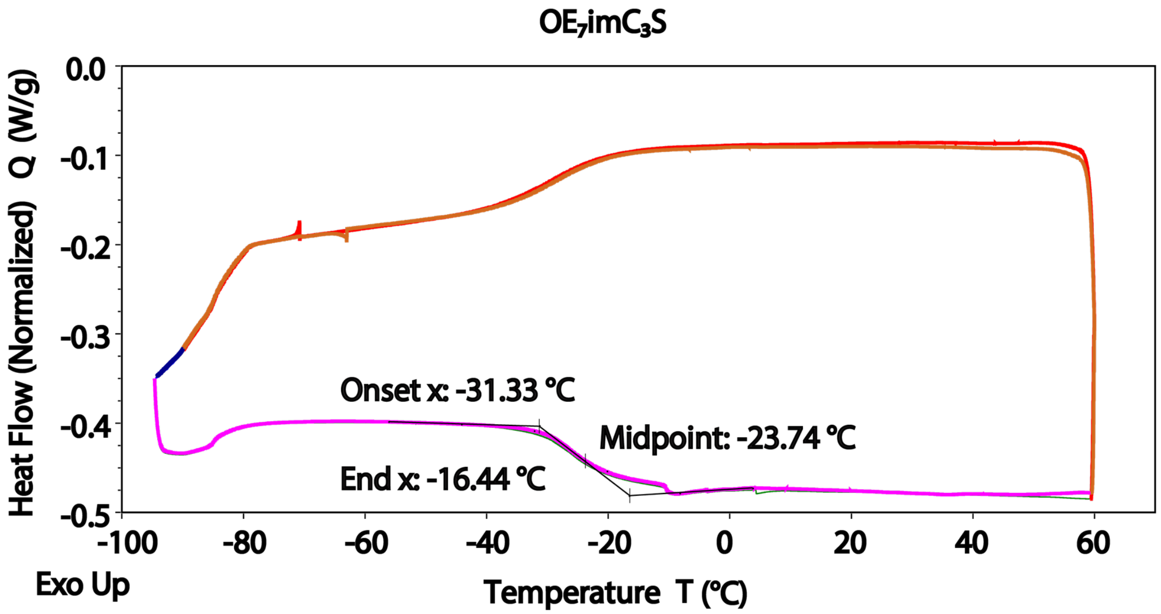

Figure S6: DSC thermogram for OE_7imC_3S.

Table S1: Experimental and simulated densities ρ ($g\,cm^{-3}$) for ZwLs.

Zwitterionic Liquids	Temperature (K)	Simulation	Experiment*
OE_2imC_3S	298	1.267	1.294
	450	1.186	
	500	1.155	
	600	1.096	
	700	1.039	
OE_3imC_3S	298	1.246	1.281
	450	1.161	
	500	1.129	
	600	1.066	
	700	1.005	
OE_7imC_3S	298	1.204	1.199
	450	1.096	
	500	1.060	
	600	0.986	
	700	0.915	

* The standard deviation associated with density measurement is estimated to be 0.005 $g\,cm^{-3}$.

Table S2: Onset glass transition temperatures (T_g) for ZwLs.

Zwitterionic Liquids	T_g (K)
OE_2imC_3S	241.15
OE_3imC_3S	244.55
OE_7imC_3S	241.82

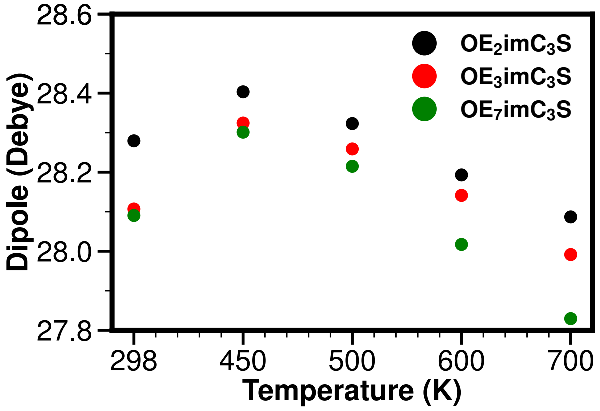

Figure S7: Average temperature-dependent dipole moments for the ZwLs showing that these do not appear to change significantly as a function of temperature or as a function of chemical composition. What determines these dipoles is the consistent length of propyl chains separating positive and negative moieties.

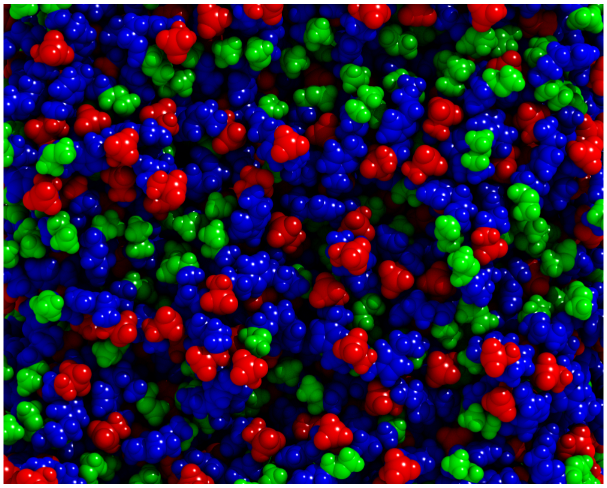

(a) OE_2imC_3S

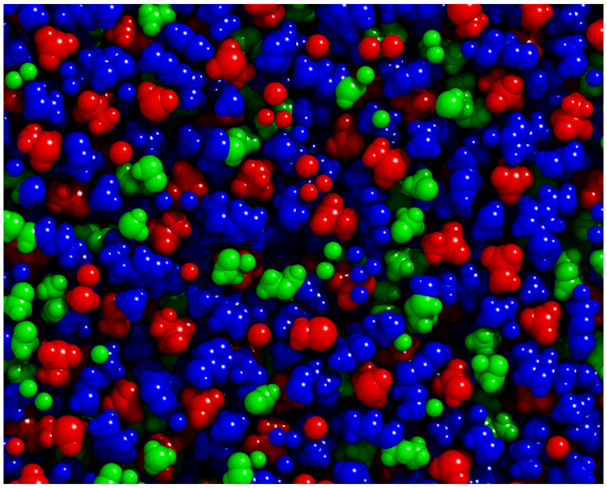

(b) $OE_2eim^+/H_3CSO_3^-$

Figure S8: Simulation snapshot for OE_2imC_3S and its IL analogue $OE_2eim^+/H_3CSO_3^-$. In the Figure, tail components are depicted in green, cationic head components in blue, and anionic components in red. Compare this plot with Figure 3 for the case of OE_7imC_3S and its IL analogue $OE_7eim^+/H_3CSO_3^-$.
